# The level and patterns of fertility among women in Kersa Demographic Surveillance and Health Research Center (KDS-HRC) Field site, Kersa District, East Ethiopia

**DOI:** 10.1186/s40738-015-0010-5

**Published:** 2015-11-17

**Authors:** Desalew Zelalem, Agumasie Semahegn, Gezahegn Tesfaye, Balewgize Sileshi

**Affiliations:** College of Health and Medical Sciences, Haramaya University, P.O. Box- 235, Harar, Ethiopia

**Keywords:** Level, Fertility, Demographic, Women, Kersa, Ethiopia

## Abstract

**Background:**

Fertility is one of the three principal components of population dynamics. High fertility and rapid population growth exert negative influences on economic and social development. This study was aimed to estimate the level and trends of fertility among (15–49 years) old women in kersa demographic surveillance and health research center, kersa district Eastern Ethiopia.

**Methods:**

The study was conducted at kersa demographic surveillance and health research center in kersa district, Eastern Ethiopia. The study utilized five years follow up data (2008–2012). All women (15 to 49 years old) who have been living at kersa demographic surveillance and health research center in kersa district from 2008 to 2012 were included in the analysis to estimate the level and pattern of fertility. Descriptive statistics was computed to determine level and pattern of fertility.

**Results:**

Age specific fertility rate was highest in women of the age group 25–29 years old (233.2 per 1000 women in 2008; 205.8 per 1000 women in 2009; 279.0 per 1000 women in 2010; 186.6 per 1000 women in 2011 and 198.5 per 1000 women in 2012) in five consecutive years from 2008 to 2012. Total fertility rate didn't show any significant decline during the study period, i.e., 4.3, 4.5, 4.9, 3.5, 4.0 live births per woman throughout her reproductive period (15–49) years old in 2008, 2009, 2010, 2011 and 2012, respectively. On the other hand, general fertility rate declined from 110.3 births per 1000 women in 2008 to 95.9 per 1000 women in the reproductive age in 2012.

**Conclusion:**

The total fertility rate was found to be relatively high. Fertility rate is higher in rural residents and illiterate women than in urban residents and literate women. Strong information, education, communication and behavior change communication on family planning should be designed and implemented to prevent unwanted fertility.

## Background

The total fertility rate (TFR) is the average number of children a woman would bear if fertility rates remain unchanged during her lifetime [1]. Fertility is one of the three principal components of population dynamics that determine the size, structure and composition of the population in any country [2]. At the beginning of the twenty first century, Sub-Saharan Africa is characterized by a high population growth rate. The population of this region in mid-2008 was around 809 million. Compared to other developing countries, fertility is highest in Sub-Saharan Africa, at an average of 5.4 children per woman [3].

In the majority of the least developed countries, the number of children that women have surpasses the number desired even though for some least developed countries the desired number of children remains higher than the achieved fertility. Suggesting that universal provision of family planning services could result in a reduction of unwanted fertility [1]. Over the next 50 years, Sub-Saharan African countries are expected to become the primary source of global population increase. Countries such as Nigeria, Congo, Uganda and Ethiopia are likely to rise quickly in the ranking of the largest contributors to population growth. Major change occurs between 1950 and 2050 in the ranking of the largest contributors to annual population change. Ethiopia which was twenty third in the ranking of the largest contributors to annual population change in 1950, ranked 8^th^ in 2002 and projected to be third in 2050. The population of Sub-Saharan Africa is growing at 3 % per year [4].

The 20^th^ century was the period in which Ethiopia experienced the most rapid population growth in its history. The population of Ethiopia in 1900 was estimated at 11.8 million and this figure has grown to 23.6 million in 1960, taking 60 years to double in 1988 aafter 28 more years, the population has grown to 47.3 million [5]. Its current population is close to 83 million [3], about a seven fold increase in the last century. Currently the population is growing at a rate of 2.6 % per annum [6] indicating that the population of the country is growing rapidly and will double in size in about 26 years if the present growth rate persist. According to the report of the central statistical agency projection based on the 2007 population and housing census results, the Ethiopian population reached 86 million people in July 5, 2013 [7]. In line with this, in about 12 years, the country will hit the 100 million mark [6, 7].

Ethiopia is one of the Sub-Saharan African countries where high and persistent fertility rate has been seen for a long period of time and ranks second in Africa next to Nigeria. Although slightly decreasing trend has been seen in fertility rate from year to year, it is still high as compared to developed nations. Various factors keeping the fertility rates high include poverty, war, famine, low level of education, economic status and less autonomy of women and traditional barriers to contraception (Tegenu T: Exponential Population Growth and Carrying Capacity of the Ethiopian Economy, unpublished; [6]).

Fertility level indicators such as crude, age specific and total fertility rates are among the highest in the world. Crude birth rate and total fertility rate are 34.5 births per 1,000 population and 4.8 children per woman, respectively. The fertility among adolescents aged 15–19 in Ethiopia is 79 births per 1,000 women. Fertility is higher in rural (5.5 children per woman) compared to urban (2.6 children per woman) areas [3]. High fertility and rapid population growth exert negative influences on economic and social development and low levels of economic and social development provide the climate favoring high fertility and hence rapid population growth [5]. Moreover high fertility is associated with increased obstetric and medical risks for mothers. Fertility is also high where maternal and infant and child mortality rates are high. On the other hand, fetal deaths, low birth weight at birth and related problems are also associated with unregulated fertility [5, 9].

The level of fertility in the country also varies by region. According to the 2011 Ethiopian Demographic and Health Survey, Oromia regional state, in which the current study was conducted, had the second highest total fertility rate (5.6 children per woman) next to Somali regional state (7.1 children per woman) in the country [3]. The population of this region has grown by 2.9 percent per annum between 1994 and 2007 censuses of the country which is higher than the national level average growth rate of 2.6 percent per annum [10]. The level of fertility varies even among districts in a region. This variation of fertility is not well assessed in the eastern Hararghe zone of Oromia regional state in general and Kersa district in particular. Moreover, most fertility assessments in the country are made based on cross sectional data and hence analysis of fertility based on data collected prospectively and on a continuous basis is limited. Thus the current study tried to assess the level and pattern of fertility at the district level using data collected prospectively and on continuous basis (Demographic surveillance data).

The contribution of fertility to changes in the size and structure of the population in Ethiopia and its link to the success of reproductive health programs and policies requiresdetailed study. Fertility has been the focus of such programs. Identifying the fertility levels and patterns enables to better understand the mechanisms by which fertility changes in a defined community with respect to the prevailing policies and programs. However, studies attempting to investigate the level and pattern of fertility in Kersa district are absent and it is this paucity of information on fertility level and pattern that motivated the initiation of the current study. The current study, by identifying the level and pattern of fertility at the district level, will help in designing appropriate strategies to effectively implement programs to prevent unwanted fertility. The findings from this study will contribute to policy-makers, program managers and others understanding of changes in the fertility level at the micro level in Ethiopia. Therefore, this study aimed to assess the level and pattern of fertility among women (15–49 years old) at Kersa Demographic Surveillance and Health research Center, Kersa district, eastern Ethiopia.

## Methods

### Study setting and study period

**Fig. 1:**
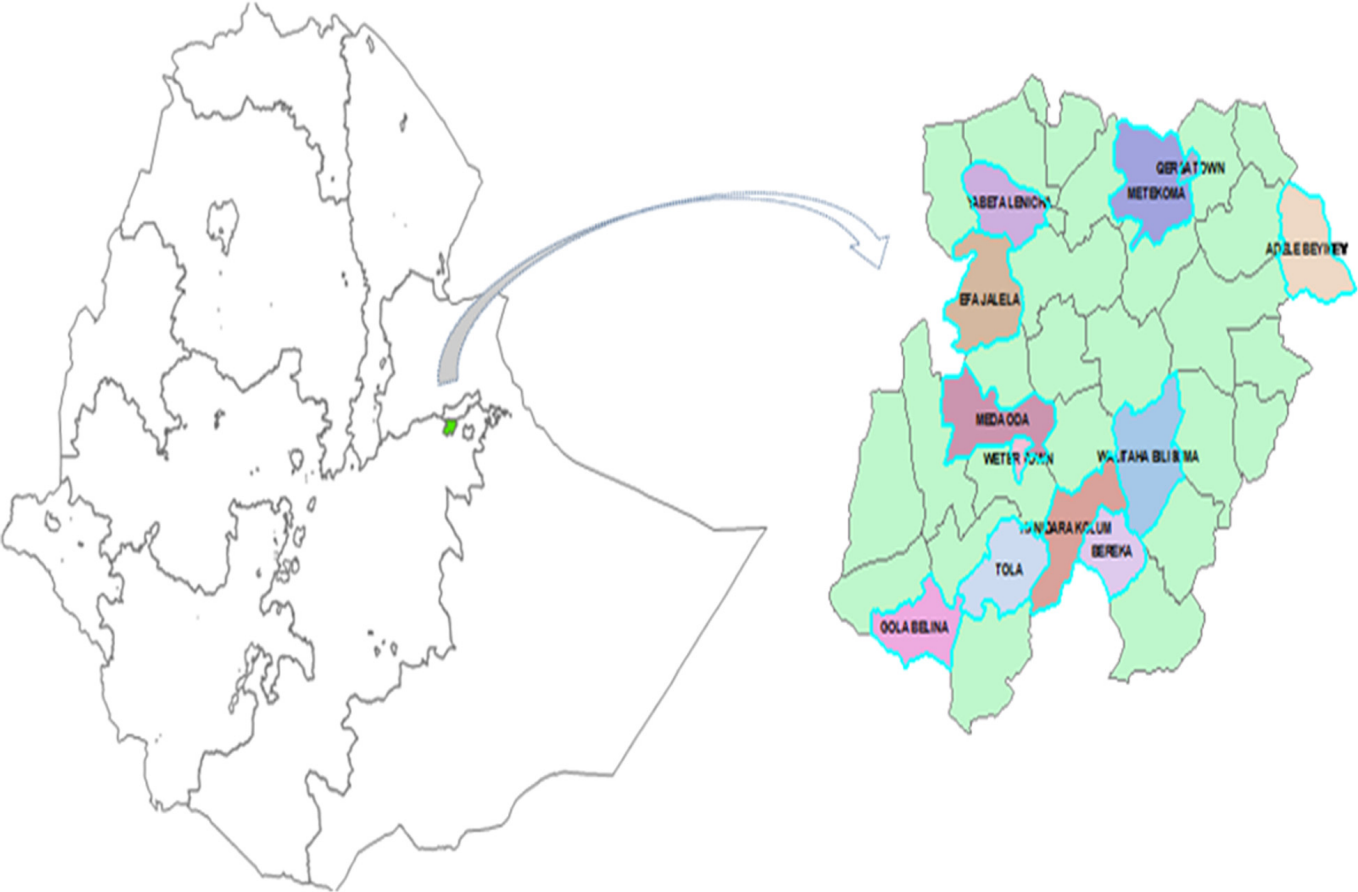
The location of Kersa Demographic Surveillance and Health Research Center in Ethiopia and the 12 Kebeles of the study site (the shaded once)

This study was part of the Kersa demographic surveillance and health research center in kersa district from 2008–2012. The surveillance site was established in September 2007 in Kersa district, Eastern Hararghe of Oromia region, East Ethiopia with the aim of tracking demographic changes like death, birth, migration and marital status change. The surveillance activities further extended by adding surveys in nutrition, reproductive health, environmental health, HIV/AIDS, morbidity, health seeking behavior and health care utilization during the months of January-March 2008. The surveillance activity was instituted in 12 kebeles (the smallest administrative unit in Ethiopia with approximate population size of 4–5 thousand) of which two are semi-urban and the remaining 10 are rural kebeles. (Fig. 1) According to the first census of kersa demographic surveillance and health research center (KDS-HRC), there were 10,256 households and 53,462 people in the study site with an average household size of 5.2 and sex ratio of 105. In the study area the crude birth and death rates were 26.8 and 9.2 per 1000 population respectively. Infant and under five mortality rates were 44.9 and 108.2 per 1000 live births, respectively.

### Study design and population

The area of a Demographic and Health Surveillance System (DHSS) site mainly depends on the size of the population required for demographic surveillance and related research activities. The size is also influenced by pragmatic considerations, such as the cost of the research center and its local capacity to manage the associated logistics and human resources. The DHSS is a set of operations that longitudinally follow well-defined entities or primary subjects (individuals, households, and residential units) and all related demographic and health related outcomes within a clearly defined geographic area. The DHSS site follows the DHSS approach and follows-up every individual within a defined catchments area four times a year with house-to-house visits. All women (15–49 years old) living at KDS-HRC in the Kersa district from 2008 to 2012 were included in our analysis to estimate the level and trend of fertility.

### Data collection procedure

The data were collected by structured questionnaires which were administered by using the appropriate local language (Affan Oromo) which wass prepared by the kersa demographic surveillance and health research center. The population and households were given their own code or identification number during baseline enumeration. The population update form was used to register the occurrence of events within each household between two visits. For each event identified in the population update form, details were recorded on the appropriate vital events registration forms.

### Data quality assurance

The data collection process was supervised by field supervisors after they underwent intensive training by the research team.Field workers were community members who were skilled in the local languages and whohad completed at least secondary school education. Trainings targeted interview skill development and were constantly provided. Field workers checked their own completed forms before they handed them over to their supervisors. The field supervisors checked the filled in questionnaires and provided feedback to the data collectors and if there were errors on the filled in forms, data collectors corrected in the field. Data entry clerks entered the data with supervision by data entry supervisors.

### Variables

Variables like age of women, age at first birth, age at first marriage, educational status, occupational status, residence, religion, marital status, live birth, number of children born alive, male and female birth, distance from health center and climatic zones were used to estimate the level and trend of fertility at the kersa demographic surveillance and health research center (KDS-HRC) in kersa district.

### Statistical analysis

The data were checked, edited in the field and at the office for incompleteness and inconsistency by field supervisors and the research team. Incomplete and inconsistent data were sent back to the field and were corrected, hence except for those women who were migrated out of the study area or died, there were no people lost to follow up during the study period. In the Kersa Demographic Surveillance and Health Research Center, people who were initially registered as members of the study site then found to be left from the study site in the subsequent visits, were registered as out-migrants. Similarly, if people who initially were not residents of the study site, were found to have arrived in the study site and lived there at least for six months, they were registered as in-migrants. So the basic characteristics of the study participants in the site were recorded immediately as they were registered as residents of the study site. Hence, there were no people lost to follow up. The detail of the Kersa Demographic Surveillance and Health Research Center methodology is available in other study [11]. Thus the response rate was considered to be 100 percent. The data were entered into the KDS data base system (kersa soft) by data clerks and exported to SPSS window version 16.0 statistical software. Final data cleaning was done by computing frequency and exploration through whisker box plot for outliers. Descriptive statistics was computed to determine the level and trend of fertility. Crude Birth Rate (CBR), Age Specific Fertility Rate (ASFR), General Fertility Rate (GFR) and Total Fertility Rate (TFR) with different sociodemographic variables were estimated to determine the level of fertility. The chi-square for trend was used to test the trend of fertility for the last five years (2008–2012).

$$ \mathrm{C}\mathrm{B}\mathrm{R}=\frac{\mathrm{number}\kern0.5em \mathrm{of}\kern0.5em \mathrm{L}\mathrm{B}\kern0.5em \mathrm{in}\kern0.5em \mathrm{year}\kern0.5em \times 1000}{\mathrm{mid}\kern0.5em \mathrm{year}\kern0.5em \mathrm{population}} $$

$$ \mathrm{ASFR}=\frac{\mathrm{total}\kern0.5em \mathrm{L}\mathrm{B}\kern0.5em \mathrm{a}\kern0.5em \mathrm{given}\kern0.5em \mathrm{a}\mathrm{ge}\kern0.5em \mathrm{group}}{\mathrm{midyear}\kern0.5em \mathrm{female}\kern0.5em \mathrm{population}\kern0.5em \mathrm{same}\kern0.5em \mathrm{a}\mathrm{ge}\kern0.5em \mathrm{group}\times 1000} $$

$$ \mathrm{T}\mathrm{F}\mathrm{R}=\mathrm{Sum}\ \mathrm{of}\ \mathrm{ASFR}\kern0.5em  \times 5\kern0.5em \mathrm{or}\ \mathrm{T}\mathrm{F}\mathrm{R}={\displaystyle \sum_{\mathrm{i}=15}^{49}\frac{{\mathrm{B}}_{\mathrm{i}}}{{\mathrm{P}}_{\mathrm{i}}^{\mathrm{f}}}}\times 1000 $$

$$ \mathrm{G}\mathrm{F}\mathrm{R}=\frac{\mathrm{number}\kern0.5em \mathrm{of}\kern0.5em \mathrm{births}\kern0.5em \mathrm{at}\kern0.5em \mathrm{same}\kern0.5em \mathrm{year}}{\mathrm{mid}\kern0.5em \mathrm{female}\kern0.5em \mathrm{population}\kern0.5em \mathrm{at}\kern0.5em \left(15\hbox{-} 49\right)\kern0.5em \mathrm{same}\kern0.5em \mathrm{year}\times 1000} $$

### Ethical consideration

Ethical clearance was secured during the establishment of the longitudinal project from the Haramaya University ethical review committee, Ethiopian public health association (EPHA) ethical review board, and the American Center for Disease Control (CDC) Atlanta. The study participants were informed about the purpose of the study and informed verbal consent was obtained during the establishment of the project, baseline and follow up data collection. The right of study subject’s was respected at any time of the interview. Information confidentiality was reserved anonymously.

## Results

At the beginning of the survey 14,655 women in the reproductive ages, 15–49 years old, and 1616 live births were registered in 2008. During the follow up survey in 2009, 15, 246 womenand 1756 live births were registered, in 201016016 women in and 1983 live births were registered. In 201117735, and 1549 live births were registered. In 2012 18456 1770 live birth were registered. Approximately three-quarters of women had their first birth before the age of 19 years old [Table 1]. From the total 7148 mothers who gave birth in the five years, the majority 6037(84.5 %) were illiterate and 1111 (15.5 %) were literate.

**Table 1:** Distribution of live births, reproductive age women and age specific fertility rates per 1000 women in Kersa Demographic Surveillance and Health Research Center, Kersa District, East Ethiopia, 2008_2012

Age	2008			2009			2010			2011			2012		
	Women	Births	ASFR	Women	Births	ASFR	Women	Births	ASFR	Women	Births	ASFR	Women	Births	ASFR
15-19	5585	123	22.0	5899	138	23.4	6233	154	24.7	7118	122	17.1	7477	135	18.1
20-24	2158	394	182.6	2187	499	228.2	2469	518	209.8	2624	398	151.7	2748	380	138.3
25-29	2007	468	233.2	2109	434	205.8	2072	578	279.0	2272	424	186.6	2277	452	198.5
30-34	1833	355	193.7	1880	413	219.7	2046	430	210.2	2188	374	170.9	2235	423	189.3
35-39	1337	180	134.6	1379	177	128.4	1318	189	143.4	1436	146	101.7	1512	234	154.8
40-44	1137	78	68.6	1186	81	68.3	1296	93	71.8	1462	77	52.7	1499	134	89.4
45-49	598	18	30.1	606	14	23.1	582	21	36.1	635	8	12.6	708	12	16.9
Total	14,655	1616		15,246	1756		16,016	1983		17,735	1549		18,456	1770	
Percentage distribution of reproductive age women by year and age category															
	N(%)			N(%)			N(%)			N(%)			N(%)		
15-19	5585(38.1)			5899(38.7)			6233(38.9)			7118(40.1)			7477(40.5)		
20-24	2158(14.7)			2187(14.3)			2469(15.4)			2624(14.8)			2748(14.9)		
25-29	2007(13.7)			2109(13.8)			2072(12.9)			2272(12.8)			2277(12.3)		
30-34	1833(12.5)			1880(12.3)			2046(12.8)			2188(12.3)			2235(12.1)		
35-39	1337(9.1)			1379(9.0)			1318(8.2)			1436(8.1)			1512(8.2)		
40-44	1137(7.8)			1186(7.8)			1296(8.1)			1462(8.2)			1499(8.1)		
45-49	598(4.1)			606(4.0)			582(3.6)			635(3.6)			708(3.8)		
Total	14,655(100)			15,246(100)			16,016(100)			17,735(100)			18,456(100)		

The age specific fertility rate was highest in the age group of 25–29 years old (233.2 per 1000 women in 2008; 205.8 per 1000 women in 2009; 279.0 per 1000 women in 2010; 186.6 per 1000 women in 2011 and 198.5 per 1000 women in 2012 one same age group of women) in five consecutive years from 2008 to 2012. Fertility observed in the two extreme ages, i.e., among adolescents (15–19) and old women (above 35 years old) was relatively small compared to other age categories. Generally, age specific fertility rate showed very slight declines starting from the year 2010 to 2012 among women of reproductive age [Table −1].

**Fig. 2:**
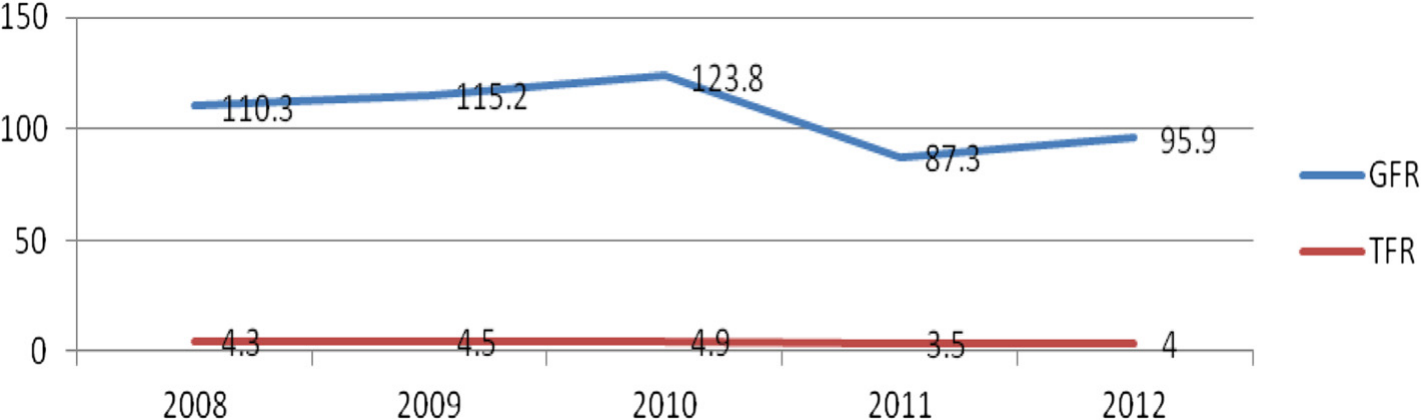
General fertility rates and total fertility rates among Women in Kersa Demographic Surveillance and Health Research Center, Kersa District, East Ethiopia, 2008_2012

There was no as such any significant decline in the total fertility rate (TFR) in the study period i.e., 4.3, 4.5, 4.9, 3.5, 4.0 live births per woman in her reproductive period (15–49 years old) in 2008, 2009, 2010, 2011 and 2012, respectively. Similarly, the decline in general fertility rate was not as such big, i.e., it declined from 110.3 births per 1000 women in reproductive age in 2008 to 95.9 per 1000 women in the reproductive age in 2012 [Fig. 2].

Approximately three fourth of women gave birth before twenty years old. Relatively higher percentage of women gave birth below twenty years old in 2009 (73.7 %) and 74.4 % in 2010. Almost one in three women give birth when their age was twenty years and above. There was no significance difference across age at first birth in the five consecutive years (2008–2012).

**Fig. 3:**
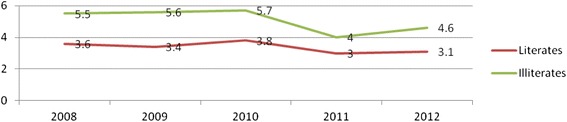
Pattern of Total Fertility Rate by literacy status of women at Kersa Demographic Surveillance and Health Research Center, Kersa District, East Ethiopia, 2008_2012

There was a difference in the total fertility rate pattern between literate and illiterate women. The total fertility rates among illiterate women were approximately two times higher than the total fertility rate of the literate women. The interesting thing here, was that there was consistent decrement of fertility among both literate and illiterate women [Fig. 3].

**Fig. 4:**
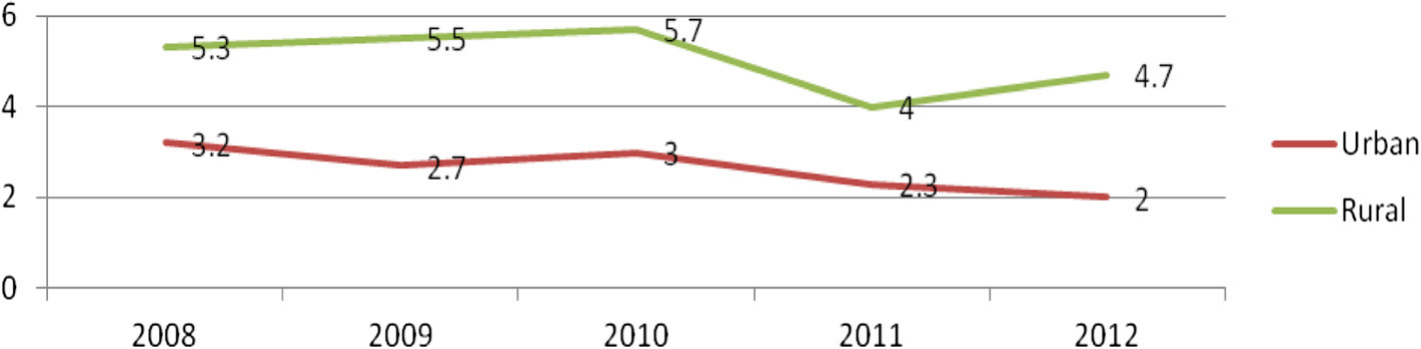
Trends of total fertility rate by rural and urban residence of women at Kersa Demographic Surveillance and Health Research Center, Kersa District, East Ethiopia, 2008_2012

The trends in TFR in the study period didn't show as such big declines which shows that the transition in fertility among the populations of the study area was very slow. The fertility transition was very rapid in urban areas of the study population especially starting from the year 2010 the level of TFR has dropped to replacement level fertility [Fig. 4].

**Fig. 5:**
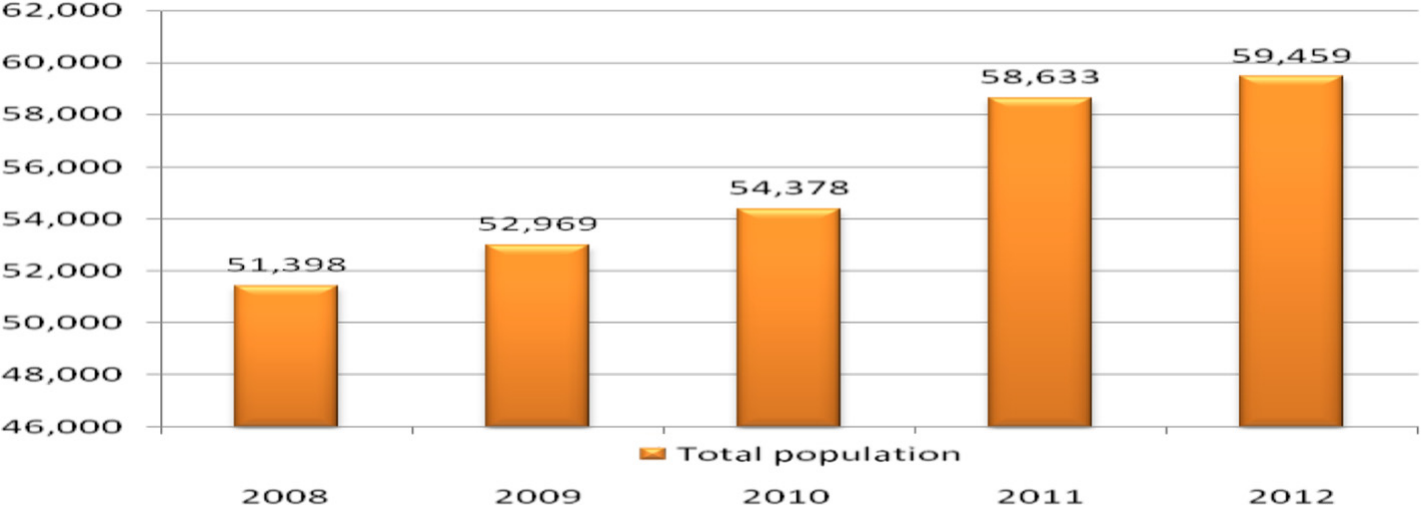
The total population of Kersa Demographic Surveillance and Health Research Center, Kersa district, East Ethiopia, 2008_2012

**Fig. 6:**
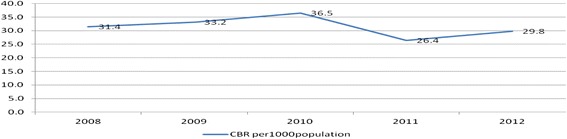
The crude birth rate of Kersa Demographic Surveillance and Health Research Center, Kersa district, East Ethiopia, 2008_2012

The population of the study area grew by 2.9 % per annum during the study period and the crude birth rate changed from 31.4 births per 1000 population in 2008 to 29.8 births per 1000 population in 2012 [Figs. 5 and 6].

## Discussion

This study determined the level and pattern of fertility rates in the Kersa demographic surveillance and health research center survey. The findings from this study showed that the population of Kersa Demographic Surveillance and Health Research Center has grown by 2.9 % per annum during the study period (i.e., from 2008_2012) and the crude birth rate has declined from 31.4 births per 1000 population in 2008 to 29.8 births per 1000 population in 2012 [Fig.s. 5 and 6]. The age specific fertility rate was highest in the 25–29 year old age group in five consecutive years from 2008 to 2012. Fertility observed in the two extreme age groups, adolescents aged 15–19 and mothers aged above 35 years was low compared to other age categories. Similarly, general fertility rates declined from 110.3 births per 1000 women in 2008 to 95.9 per 1000 women in the reproductive age in 2012. Generally, age specific fertility rates, general fertility rates and total fertility rates (TFR) declined from 2008 to 2012 among women of reproductive age.

The TFR in this study declined on average by 0.19 % annually. Although a change in TFR over five years was not statistically significant (P = 0.4134; 95 % CI −0.6975729, 0.3775728) the direction of change was declining. Absence of a statistically significant change in TFR could be because of shortness of the study period, i.e., only covering five years in which significant changes in factors affecting fertility may not be expected. The decline in TFR observed in this study (from 4.3 in 2008 to 4.0 in 2012) is comparable with findings from Kenya in the years 2002–2008 in which TFR declined from 4.92 to 3.92 [12]. This pattern is also consistent with the fertility situation observed in three consecutive Demographic and Health Surveys in Ethiopia in which TFR has declined from 5.5 in 2000 to 5.4 in 2005 and then to 4.8 in 2011 [3, 13, 14].

The level of TFR in this study is higher than the TFR of Namibia in 2006 which was 3.6 births per woman [15], Zimbabwe in 2005/06 in which TFR declined to less than four births [16], TFR of United states of America in 2009 which was 2.01 [17, 18] and the TFR of South Africa in 2003 which was 2.9 births per woman [19] but it is comparable to the fertility rates of some districts in South Africa in 2006 i.e., Tambo district (TFR = 4.1), Greater Sekhukhune district (TFR = 3.7), UThukela district, Alfred Nzo district and Vhembe district (TFR = 3.6), Zululand district and Capricon district (TFR = 3.4) and UMzinyathi district (TFR = 3.4) [20]. However, the level of TFR observed in this study is lower than the levels of TFR observed in Uganda and Tanzania which were 6.7 and 5.7 children per woman during their most recent surveys [21]. According to the UN estimation of total fertility rates for the period 1960 to 2000 based on Demographic and Health Survey data, a smooth decline has been observed in Mali, Mauritania, Niger, Guinea, Senegal, Burkina Faso, Gambia and transition to lower rates was achieved by different countries of the world, particularly in sub-Saharan African and Asian countries [22, 23].

Age specific fertility rate was highest in the 25–29 years old age group in five consecutive years from 2008 to 2012. Age specific fertility rates observed in the two extreme ages. i.e., for adolescents aged (15–19) and for mothers aged > =35 years was lower compared to other age categories. The findings of this study are comparable to the findings of other studies. For example, there was high age specific fertility rate for women 20–24 years old in Zimbabwe, and in other similar studies fertility peaks for ages 25–29 years old both in rural and urban areas [16]. The finding of this study is also in line with the Ethiopian Demographic and Health survey in which almost twelve percent of adolescents (15–19) gave birth and the age specific fertility rate for this age group was 79 births per 1,000 women [3]. The teenage fertility observed in this study was much higher than teenage fertility in United States of America in which age specific fertility rate for women aged 15–19 years in 2003 was 41.6 births per 1,000 [24].

Age specific fertility rates, total fertility rates and general fertility rates were higher among rural resident women than urban residents. Similarly, illiterate women had higher fertility rates than literate women. This finding is also apparent in the results in Namibia, that women who live in rural areas have more children than those in urban areas. The effect of place of residence on the total number of children ever born indicates a decrease from 1992 to 2006. In the long run it is anticipated that fertility will decrease among women in the rural areas [15]. In South Africa, a survey conducted in 2003 indicated that 2.3 % of women aged 15–19 years were pregnant with their first child, and the fertility rate was higher in rural (2.8) than in urban (2.1) areas [19]. This study shows lower rates than studies performed in Uganda, Kenya, Zimbabwe and Tanzania in terms of the fertility rate, but shows similar fertility by residence difference in which fertility is higher in rural area than urban areas [21]. This scenario is similar in the Ethiopian demographic and health survey 2011 which was The GFR in Ethiopia is 161 live births per 1,000 women of reproductive age;e rate is considerably higher in rural areas (184) than in urban areas [3].

### Implications of the finding

Ethiopia is the second populous country in Africa with a growth rate of 2.6 percent per year and total fertility rate of 4.8 children per woman. The Federal Ministry of Health has strived to achieve the MDG number 4 and 5. The goal of the country is to reduce TFR to 4.0 through education of women and universally accessibility of family planning to women in the reproductive ages. Therefore, findings of the current study have clear implications for policy makers, program planners and any concerned bodies in their efforts to design strategies to tackle unwanted fertility.

### Strength and limitation of the study

The strength of this study is that it used community based quantitative demographic and health surveillance data for five consecutive years. However, the data are self reported so the accuracy of the response depends on the willingness of the respondent to give trustworthy information. This is a cross sectional study design that can not establish cause and effect relationship.

## Conclusion

Total fertility rate was declining. Fertility rate is higher in rural residents and illiterate women than urban residents and literate women. Strong information, education, communication and behavior change communication on family planning, and should be designed and implemented to prevent unwanted fertility.
